# Carry-over coarticulation in joint angles

**DOI:** 10.1007/s00221-015-4327-4

**Published:** 2015-05-24

**Authors:** Eva Hansen, Britta Grimme, Hendrik Reimann, Gregor Schöner

**Affiliations:** Institut für Neuroinformatik, Ruhr University Bochum, Bochum, Germany; Department of Kinesiology, Temple University, Philadelphia, PA USA

**Keywords:** Motor control, 3D human arm movements, Coarticulation, Joint angles, Uncontrolled manifold, Motor equivalence

## Abstract

Coarticulation indicates a dependence of a movement segment on a preceding segment (carry-over coarticulation) or on the segment that follows (anticipatory coarticulation). Here we study coarticulation in multidegrees of freedom human arm movements. We asked participants to transport a cylinder from a starting position to a center target and on to a final target. In this naturalistic setting, the human arm has ten degrees of freedom and is thus comfortably redundant for the task. We studied coarticulation by comparing movements between the same spatial locations that were either preceded by different end-effector paths (carry-over coarticulation) or followed by different end-effector paths (anticipatory coarticulation). We found no evidence for coarticulation at the level of the end-effector. We found very clear evidence, however, for carry-over, not for anticipatory coarticulation at the joint level. We used the concept of the uncontrolled manifold to systematically establish coarticulation as a form of motor equivalence, in which most of the difference between different movement contexts lies within the uncontrolled manifold that leaves the end-effector invariant. The findings are consistent with movement planning occurring at the level of the end-effector, and those movement plans being transformed to the joint level by a form of inverse kinematics. The observation of massive self-motion excludes an account that is solely based on a kinematic pseudo-inverse.

## Introduction

In voluntary movements of daily life, we typically have more mechanical degrees of freedom (DoF) at our disposal than strictly needed. For instance, an object can be translated within the three dimensions of space with a minimum of three DoF, while the human arm has ten DoF (where we include the sternoclavicular joint). Redundancy in movement systems has been a topic of the scientific study of human movement dating back at least to Bernstein (1967) who asked how the nervous system harnesses the many DoF to achieve a particular movement goal. This led to questions about how movements observed at the joint level may be compared to their description at the end-effector level. A direct comparison is not meaningful as these levels have different metrics and dimensionality. The concept of the uncontrolled manifold (UCM) links the two levels by identifying the manifolds in joint space that map onto equivalent states at the level of the end-effector (Scholz and Schöner 1999).

The concept has been used to devise a method of analysis of the variance of movements. The hypothesis is that variation from trial to trial lies primarily in those directions in joint space along which the end-effector state does not vary, reflecting neural mechanisms to stabilize task performance that is assessed at the level of the end-effector. The Jacobian, from which these subspaces are derived, links changes in joint space to changes in the end-effector. Empirical evidence has supported this hypothesis in numerous tasks and movement systems (Scholz and Schöner 1999; Scholz et al. 2000; Reisman et al. 2002; Mattos et al. 2011; Reisman and Scholz 2006). Analogous ideas were developed to study redundancy at the muscular level (Krishnamoorthy et al. 2003) and at the level of isometric finger forces (Latash et al. 2001).

Another facet of redundancy is its role to achieve movement goals under different conditions. Although the notion of motor equivalence (MEQ) is used in a variety of meanings, one influential idea is that a redundant movement system may reach the same movement goal with different joint configurations under different conditions. The same problem as in an analysis of variance arises. However, how may we compare the degree to which a motor goal at the end-effector level is reached with the difference between different solutions at the joint level? The UCM concept can be used to address this problem. Mattos et al. (2011), for example, placed elastic bands across the elbow joint in a reaching task and varied the stiffness of the bands in different conditions. They analyzed the mean difference in joint configurations between these different conditions and found that more of that difference lies in the UCM, within which the end-effector is invariant, than orthogonal to the UCM. We call this application of the UCM concept to differences across conditions the method of MEQ.

Where does the observed UCM structure of variance and MEQ structure of systematic difference come from? Ultimately, neural networks generate motor commands and control their execution. One account (Martin et al. 2009) proposes that movement plans are generated by these neural networks at the end-effector level and are coupled to the downstream neural networks so as to preferentially stabilize motor commands within the subspace that is consistent with the end-effector plan [see Guenther et al. (1994) for a neural processing framework that may encompass this account].

Movement context provides a natural way to probe these ideas. Natural movements are embedded in sequences of motor actions. For any given movement, the preceding movement sets initial conditions for both the end-effector and the joint level, while the subsequent movement is typically thought to be specified only at the level of the task, the end-effector. Does this difference reveal itself as a difference in MEQ?

The influence of movement context runs under the label of coarticulation. In carry-over coarticulation, a movement is influenced by its predecessor, and in anticipatory coarticulation, it is influenced by its successor. Coarticulation is primarily known in speech production. The speech articulatory apparatus is redundant, and all DoF are coordinated to achieve speech (Schöner et al. 2008). Speech consists of sequences of temporal overlapping sub-movements produced at a high rate. Adjacent phonemes are interdependent and mutually influence their respective articulatory states (Fowler and Saltzman 1993). Much less is known about coarticulation in limb movements (Grimme et al. 2011). Carry-over coarticulation in human arm movements was found in a sequential obstacle avoidance task (van der Wel et al. 2007). A movement segment in which an obstacle was avoided was followed by movements that did not face obstacles. The elevation of the movement path remained higher over a number of subsequent movements than when no obstacle had been avoided. This coarticulation effect scaled with obstacle height and was observed even when the hand performing the movement switched after the avoidance movement. They also found a small anticipatory coarticulation effect, in which an upcoming obstacle led to increased path height in the movement that did not face an obstacle. Both effects suggest context dependence that derives, at least in part, from the end-effector level.

Small anticipatory coarticulation was found in typing movements (Soechting and Flanders 1992) and in playing the piano (Engel et al. 1997). Klein Breteler et al. (2003) investigated anticipatory coarticulation in 3D drawing sequences at the level of the end-effector and the plane formed by the shoulder, elbow and hand. Movements were performed to the same first target, but to different final targets. The authors did not find coarticulation at the level of the spatial path of the hand, but observed anticipatory modifications of the inclination of the plane that defines how high the elbow is lifted. Anticipatory coarticulation in limb motor control appears primarily to reflect the efficiency of the movement and the comfort of the end state (Rosenbaum et al. 2006). Generally, requirements of the task may influence movements from the very beginning even if the task is more directly linked to a late phase of the action (Ansuini et al. 2006; Marteniuk et al. 1987). For example, Marteniuk et al. (1987) compared grasping of a fragile object to grasping a soft, resilient object, to grasping a disk that will then be either thrown into a large box or fit into a tight niche. The main deceleration phase of the arm trajectory increased with the precision needed.

In this study, we look afresh at coarticulation in arm movements and use the UCM concept to learn about the level at which movement context matters. We ask participants to move an object sequentially to two locations. We observe the movement at the level of ten joint angles that are reconstructed in anatomically correct form. We examine whether different movement contexts affect the end-effector path or the joint configuration used to generate the end-effector path. To this end, we decompose the mean difference vector between joint configurations observed in different movement contexts into their UCM and their orthogonal components. We expect no or little anticipatory coarticulation, as the task does not require participants to attend to the second target before starting the sequence. We expect, however, carry-over coarticulation. If carry-over coarticulation occurs at the end-effector level, then this reflects interaction between task-level movement plans that cannot be easily attributed to end-state comfort. If carry-over coarticulation only occurs at the joint level, then this is consistent with the notion that movement plans are made at the task or end-effector level and motor equivalent solutions result from the neural networks that generate and control movement.

## Methods

### Participants

Six female and four male healthy, right-handed (determined by self-report) participants between 20 and 30 years including two of the authors participated in this study. Each gave written informed consent after a detailed explanation of the task. Apart from the authors, all participants were unaware of the purpose of the experiment.

### Experimental setup

**Fig. 1:**
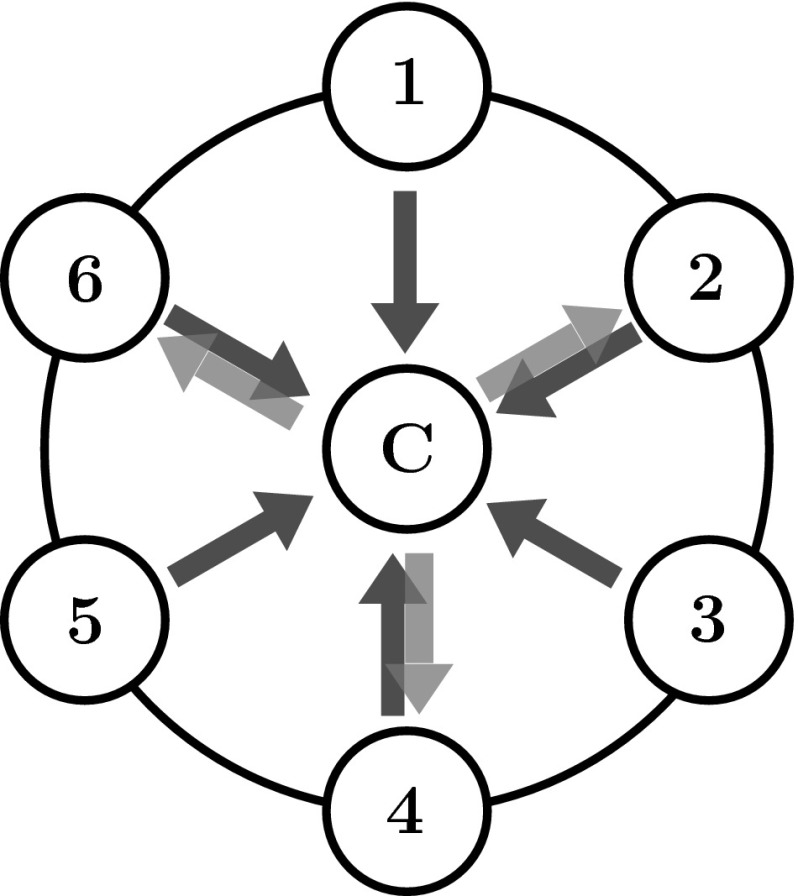
Setup of the experiment. Participants moved a cylindrical object from one of the outer starting positions (*1*–*6*) to the central target position (*C*) (first sub-movement, *red arrows*) and from there back to one of three possible final targets numbered with *2*, *4* and *6* (second sub-movement, *black arrows*)

Ten participants performed a simple sequential movement task on a monitor table, on which starting and target positions were marked as circular disks of 6 cm diameter. Six locations arranged on an imaginary circle and a center location played the role of starting and target positions (Fig. 1). The six locations on the circle had a distance of 15 cm from the center target as well as from its two neighboring targets (measured from the midpoints of the targets). The movement started from one of six outer locations. In each trial, a cylinder, whose diameter matched the target disks, had to be moved from the starting position (S1 to S6) to the target at the center (C) and from there to one of three final target positions (T2, T4 and T6) that were a subset of the starting positions (Fig. 1). The resulting 18 (six possible starting locations $\times$ three possible final targets) experimental conditions were performed in ten repetitions each in pseudo-random order. Each condition can be identified by the index of the starting position and the index of the final position, e.g., “S1–T4.” Comparisons of conditions are denoted by pairs of conditions separated by “/.” For example, when we compare the condition of starting from location 1 and terminating on target 6 to the condition starting from location 2 and terminating on target 6, we write “S1/S2–T6.”

The participants sat on a chair in front of the monitor table and were positioned centrally with respect to the center target position. To prevent participants from moving their torso, they were secured to the chair with a harness, still allowing normal scapular motion. The lightweight cylinder was made out of Styrofoam and a small wooden middle section. It had a diameter of 6 cm and a height of 15 cm.

Before the beginning of the experiment, the participants were instructed to perform movements by lifting the object slightly and positioning it as accurately as possible at a comfortable speed on the target disk. At the beginning of each trial, the central target was shown in gray color. The participants were instructed to position the cylinder on the center target and keep the grasp of the cylinder invariant across the trial. This prevented any dependency of the grasp on the starting position. Only after participants held the cylinder at the center location did one of the six starting locations light up as a green disk. After the participant had moved the cylinder to that starting location, the central target lit up in yellow and a final outer target lit up in red. The participant moved the cylinder from the starting target to the central target, briefly tapping the cylinder on that target, and then moved on to the final target, where they sat the target down. Thus, each action consisted of two movements: the first from the starting position to the central target and the second from the central target to the final target.

### Data collection and processing

Movements were recorded with the Visualeyez (Phoenix Inc.) motion capture system VZ 4000. Three trackers, each equipped with three cameras, were mounted on the wall 1.5 m above the working surface in front of the monitor table as well as to the right of and behind the participants such that the monitor table and the right arm of the participant were in view of the camera systems. Three active markers (infrared light-emitting diodes—IRED) were attached to four rigid bodies. These were placed on the four segments of the right arm: (1) slightly to the left of the acromion process to acquire clavicle/scapula motion, (2) on the lateral part of the upper arm near the elbow joint, (3) on the dorsal side of the forearm near the wrist joint and (4) on the dorsal side of the hand. Additionally, one single marker was fixed slightly over the sternum notch and served as a reference for joint angle computation. A wireless IRED marker was attached to the top of the object. The trajectories of all markers were recorded in three Cartesian dimensions at a sampling rate of 100 Hz based on a reference frame anchored on the table. The central target position was taken as the origin of each trajectory, i.e., (0, 0, 0) in 3D Cartesian space: $x$ = horizontal, $y$ = depth, $z$ = vertical. All time series were filtered by using a second-order zero-phase forward and reverse Butterworth filter with a cutoff frequency at 5.5 Hz. After filtering, movement onset and movement termination were estimated from the trajectory of the object IRED based on tangential velocity, acceleration and distance to the starting position. Trajectories that were only recorded partially due to occluded markers or other errors were eliminated (8.8 %). All trajectories were time warped so that time is measured in percent of mean movement time.

For the further analysis, we rotated the coordinate frame in which end-effector trajectories were represented, such that the new $y$-axis pointed from the starting position to the central target (for the first movement) and from the central target to the final target (for the second movement). The new $x$-axis captured movements orthogonal to this direction within the monitor plane. The $z$-axis was unchanged, capturing elevation over the monitor plane.

### Joint angle computation

We established a biomechanical model of the upper extremity with three DoF at the sternoclavicular joint, three DoFs at the shoulder joint, one DoF at the elbow, one DoF at the radioulnar joint and two DoFs at the wrist joint, for a total of ten DoFs. The marker at the sternum was used as a reference for all calculations.

The joint centers of rotation (CoR) were estimated from calibration movements that consisted of shoulder protraction/retraction and elevation/depression (sternoclavicular joint), shoulder flexion/extension and abduction/adduction (shoulder joint), elbow flexion/extension (elbow joint), pronation/supination of the lower arm (radioulnar joint) and wrist flexion/extension and abduction/adduction (wrist joint), with five repetitions each. The CoR of the shoulder and wrist joints were estimated using the symmetrical CoR estimation algorithm (Ehrig et al. 2006, SCoRE). For the CoR of the sternoclavicular joint, a simple least-squares sphere fit was used because it reduced the estimation error compared to the SCoRE method. Similarly, the axis of elbow flexion extension was estimated by the normal of a plane that optimally fit the trajectories of the lower arm markers during the calibration movement in the least-squares sense.

To obtain the joint angles from the positions of the markers, we applied a global optimization method that minimized the summed squares of the distances between the measured marker positions and the positions reconstructed from the joint angles and the biomechanical model (Lu and O’Connor 1999). The corresponding nonlinear optimization problem was solved in MATLAB using a generic gradient descent algorithm. The initial value was determined by calculating the joint angles from rotation matrices using standard techniques (Söderkvist and Wedin 1993). The remaining error between the measured and reconstructed marker positions was small (mean $\pm$ SD, $2.27 \pm 1.46\,\hbox {mm}$ for the three markers on the hand).

### Uncontrolled manifold and motor equivalence

To understand coarticulation effects in joint space, we analyzed the joint angle data with respect to the UCM of the Cartesian position of the object. This tests the hypothesis that coarticulation is more strongly visible in the null-space of the object position than in object position itself. The signature of that hypothesis is a MEQ effect in the second movement segment when comparing joint trajectories from trials with different first movement segments. The MEQ effect is established by computing the mean difference vector between joint configurations of the two conditions at matching points in time and projecting that difference vector onto the null-space of the Jacobian and its orthogonal complement. The length of the two projections per DoF is compared. More difference in the null-space than in the orthogonal space confirms the hypothesis.

At a given movement time $t$, for each participant, the mean difference, $\Delta \theta (t)$, of joint configurations between two conditions, C1 and C2, is computed from the mean joint configurations, $\overline{\theta }^{(\hbox {C1})}(t)$, and $\overline{\theta }^{(\hbox {C2})}(t)$:

1 $$\begin{aligned} \Delta \theta (t) = \overline{\theta }^{(\hbox {C1})}(t) - \overline{\theta }^{(\hbox {C2})}(t) \in {\mathbb {R}}^{10} \end{aligned}$$

The projections of this difference vector onto the null-space parallel to the UCM, $\Delta \theta _\Vert (t)$, and onto its orthogonal complement, $\Delta \theta _\perp (t)$, can be computed as

2 $$\begin{aligned} \Delta \theta _\Vert (t)= E_\Vert (t) E_\Vert ^T (t) \Delta \theta (t) \end{aligned}$$

and

3 $$\begin{aligned} \Delta \theta _\perp (t) = E_\perp (t) E_\perp ^T (t) \Delta \theta (t), \end{aligned}$$

based on the projection matrices $E_\Vert (t) \in {\mathbb {R}}^{10 \times 7}$ and $E_\perp (t) \in {\mathbb {R}}^{10 \times 3}$. These consist of the orthonormal vectors that span the linearized UCM and its orthogonal complement and were determined by singular value decomposition of the Jacobian matrix

4 $$\begin{aligned} J (t) = \frac{\partial }{\partial \theta } \left( {\mathbf {p}} \biggl ( \frac{\overline{\theta }^{(\hbox {C1})}(t) + \overline{\theta }^{(\hbox {C2})}}{2}(t) \biggr ) \right) \end{aligned}$$

computed from the Cartesian object position ${\mathbf {p}} \in {\mathbb {R}}^3$. We evaluated the Jacobian at the average joint configuration across the two conditions. The Jacobian varies very little across the two conditions, so that the configuration around which the Jacobian is computed has no discernible influence.

We measured the magnitude of the difference vectors within each subspace by taking the norm of the projected differences and normalizing by the dimensionality of the subspace, 7 for the UCM and 3 for the orthogonal space:

5 $$\begin{aligned} d_\Vert= & {} \frac{1}{7} \Vert \Delta \theta _\Vert \Vert , \end{aligned}$$

6 $$\begin{aligned} d_\perp= & {} \frac{1}{3} \Vert \Delta \theta _\perp \Vert . \end{aligned}$$

### Statistical analysis

We analyzed the significance of carry-over coarticulation at the level of end-effector trajectories and of joint angle configurations by employing multivariate analysis of variance (MANOVA) in MATLAB. Alpha was set at $P = 0.05$. Comparisons of the two components within and orthogonal to the UCM were based on repeated measures analysis of variance (ANOVA) with SPSS. Single means for each participant for each condition were calculated and entered into a repeated measures analysis of variance (ANOVA). The P values were adjusted for multiple comparisons using Bonferroni correction. Data values are reported as mean $\pm$ standard error (SE). Alpha was set at $P = 0.05$. The dependent variables for each subject are always the respective conditions and the repetitions. For each comparison of two conditions along or orthogonal to the UCM, the independent variables are the components of the vector between the two means, represented in the UCM basis, which is given by the singular value decomposition (see above).

Moreover, for this comparison to be meaningful, the UCM has to be similar for each movement. We verified this by calculating the angle $\gamma$ between the linearization of each UCM. This angle was small for all comparisons ($\overline{\gamma }= 0.034$; $\hbox {SD}=\pm 0.020$; maximum value = 0.17).

## Results

### Movement time and peak velocity

All end-effector trajectories consisted of two sub-movements. The first one started from one of six possible initial positions and terminated at the central target, whereas the second sub-movement started from the central target and aimed at one of three possible final target positions. The first sub-movement took $0.65\,\hbox {s}\,(\hbox {SE}=\pm 0.11$) and the second sub-movement $0.59\,\hbox {s}\,(\hbox {SE}=\pm 0.09$). The velocity peak was $0.33\,\hbox {m s}^{-1}\,(\hbox {SE}=\pm 0,056$) for the first and $0.29\,\hbox {m s}^{-1}\,(\hbox {SE}=\pm 0.022$) for the second sub-movement.

### Uncontrolled manifold (UCM) variance analysis

The two components of joint configuration variance within (UCM) and orthogonal (ORT) to the UCM were analyzed for all 18 conditions for both sub-movements. To trace the time course of variance within each movement segment, we sampled each segment homogeneously (1, 25, 50, 75 and 100 %). For the first sub-movement, we performed a repeated measures ANOVA with the three main factors time, conditions and UCM/ORT. The UCM component of variance was significantly larger than the orthogonal component ($F_{1,9}=16.561, P = 0.003$). A significant interaction was detected between time and UCM/ORT ($F_{4,36}=9.872, P < 0.001$). A separate repeated measures ANOVA for each of the five points in time found, however, that the UCM component significantly exceeded the ORT component at all times.

**Fig. 2:**
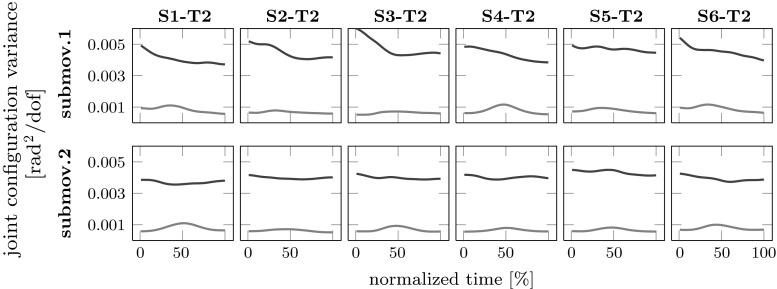
UCM results in movements from each of the six starting positions S1–S6 (column 1–6) to the final target T2 for the first and the second sub-movement (row 1–2). In each case, the component of joint configuration variability per DoF that lies parallel to the UCM (UCM, *black line*) is distinctly higher than the component that lies orthogonal to the UCM (ORT, *red line*). The results are averaged over repetitions and participants

Similarly, the repeated measures ANOVA for the second sub-movement revealed that the UCM component significantly exceeded the ORT component ($F_{1,9}=16.406, P = 0.003$). We found the same interaction between the main factors time and UCM/ORT ($F_{4,36}, P = 0.002$). Repeated measures ANOVAs for each of the five points in time showed again that the UCM component always significantly exceeded the ORT component. To illustrate the strong UCM effect, we show in Fig. 2 the UCM and ORT components of variance for target T2 across both sub-movements and the six conditions.

### End-effector trajectories

**Fig. 3:**
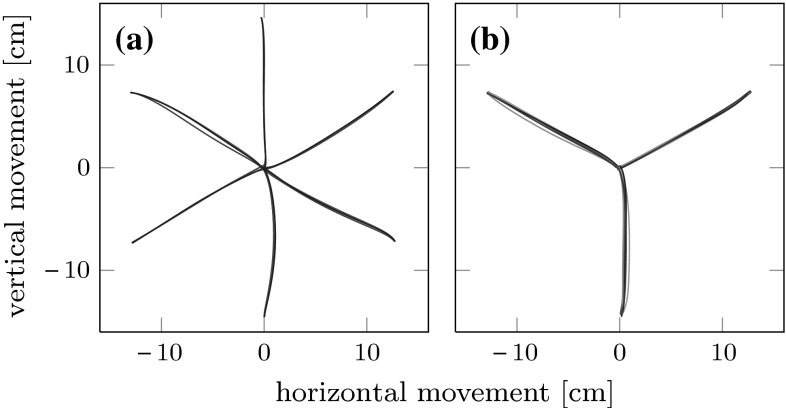
Object paths (mean over all repetitions and over all participants) projected onto the table plane. **a** Movement paths of the first sub-movement from the six starting points (S1–S6) to the central target C, but going to three different final targets are overlaid. First sub-movement paths belonging to different second sub-movement paths differ very little. **b** Movement paths of the second sub-movement from the central target C to the three final targets, but coming from the six different starting positions are overlaid. Second sub-movement paths belonging to different first sub-movement paths differ very little

**Fig. 4:**
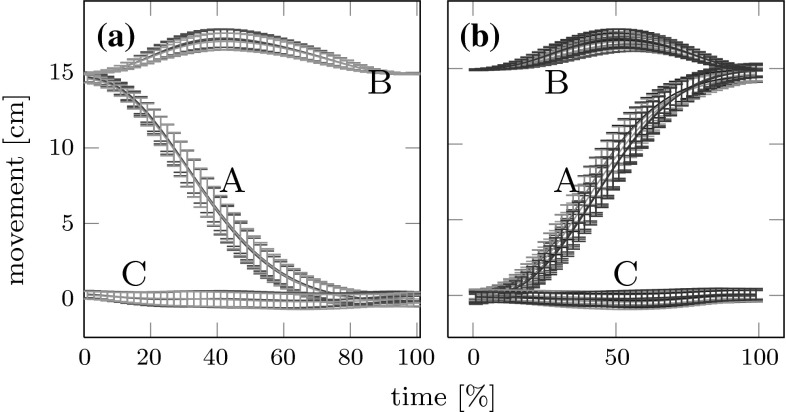
**a** Averaged end-effector trajectories with error bars representing standard deviations are overlaid for the first sub-movement in three conditions, S1–T2/T4/T6. **b** Same for the second sub-movement in six conditions, S1/S2/S3/S4/S5/S6–T2. In both cases, the three Cartesian components are plotted (*A* coordinate axis pointing from initial position to the target, *B* coordinate axis pointing up vertically, *C* coordinate axis orthogonal to *A* and *B*)

To analyze the effects of carry-over coarticulation, we compared the second sub-movements of the end-effector trajectories going to the same final target position (T2, T4 or T6) but coming from different starting positions (S1, S2, S3, S4, S5 and S6). In Fig. 3b, the two-dimensional end-effector paths for the second sub-movement to the three final targets are plotted on top of each other across the different starting locations. For each starting location, the plotted path is averaged across repetitions and subjects. The figure suggests that the end-effector paths of the second sub-movement are only very slightly affected by the preceding movement. This picture therefore reveals only very little coarticulation. In Fig. 3a, we plot the first sub-movements coming from the six starting positions on top of each other across the three final targets (T2, T4 and T6). Again, there is no obvious dependency of the first sub-movement on the subsequent movement target, excluding anticipatory coarticulation. To further explore this, we plot in Fig. 4 the three Cartesian components of the end-effector trajectories with error bars representing standard deviations as functions of time. In panel (a) of the figure, we overlay trajectories of the first sub-movement that will be followed by three different subsequent movements (S1–T2/T4/T6). In panel (b), we overlay trajectories of the second sub-movement with six different preceeding movements (S1/S2/S3/S4/S5/S6–T2). Clearly, the differences across preceding or subsequent movement segment are small, lying within a standard deviation. This is true also for other possible comparisons not included in the figure.

### Joint angles of the arm with ten degrees of freedom

**Fig. 5:**
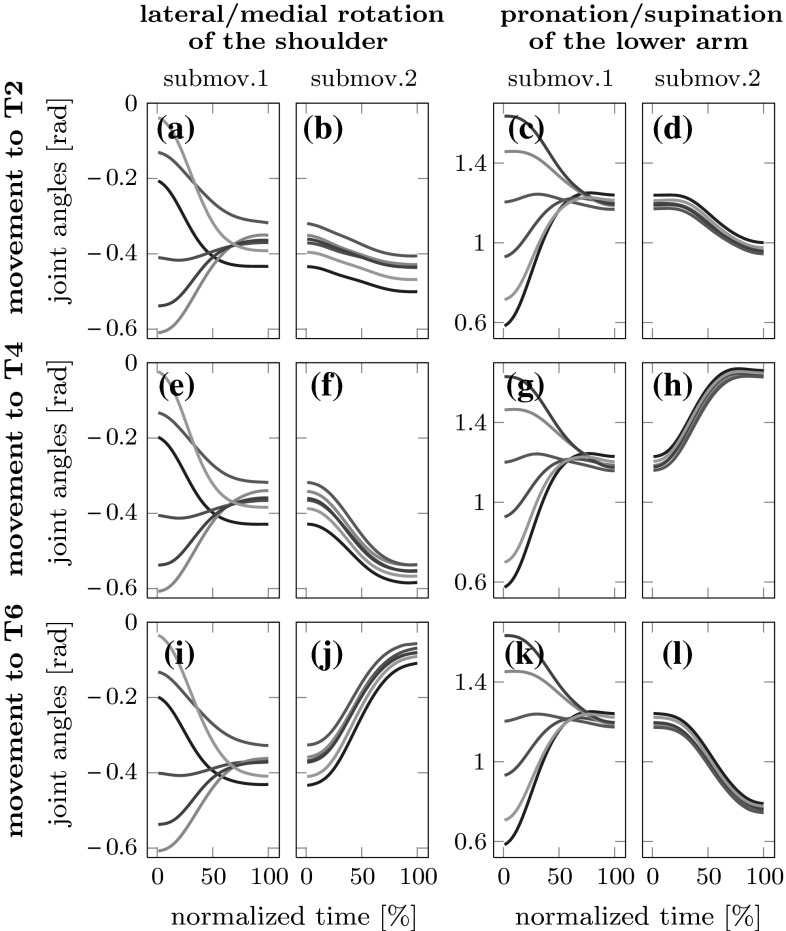
Trajectories of two joint angles, lateral/medial rotation of the shoulder (*left two columns*) and pronation/supination of the elbow (*right two columns*) plotted for movements originating at the six starting positions to the central target (sub-movement 1, *first* and *third column*) and from there to final target T2, T4 and T6 (sub-movement 2, *second* and *fourth column*) are shown. The starting positions are color coded: *blue* starting position number S1, *red* S2, *olive* S3, *gray* S4, *magenta* S5, *green* S6

To illustrate the pattern embedded in the joint angle trajectories, we first look at typical time courses of joint angles within each sub-movement. Figure 5 shows two examples of joint angles: the lateral/medial rotation of the shoulder and the pronation/supination of the lower arm. The time courses of the other eight joint angles are very similar. We overlay these trajectories for movements originating from different starting positions going to the same target position for both sub-movements. Naturally, early in the first sub-movement, joint angles differ for different starting positions. By the end of the first sub-movement, these differences have decreased, but a characteristic pattern of differences remains. Over the second sub-movement, this pattern is preserved. This suggests that at the level of joint angles, the second sub-movements are realized differently for movements coming from different starting positions, an instance of carry-over coarticulation.

**Fig. 6:**
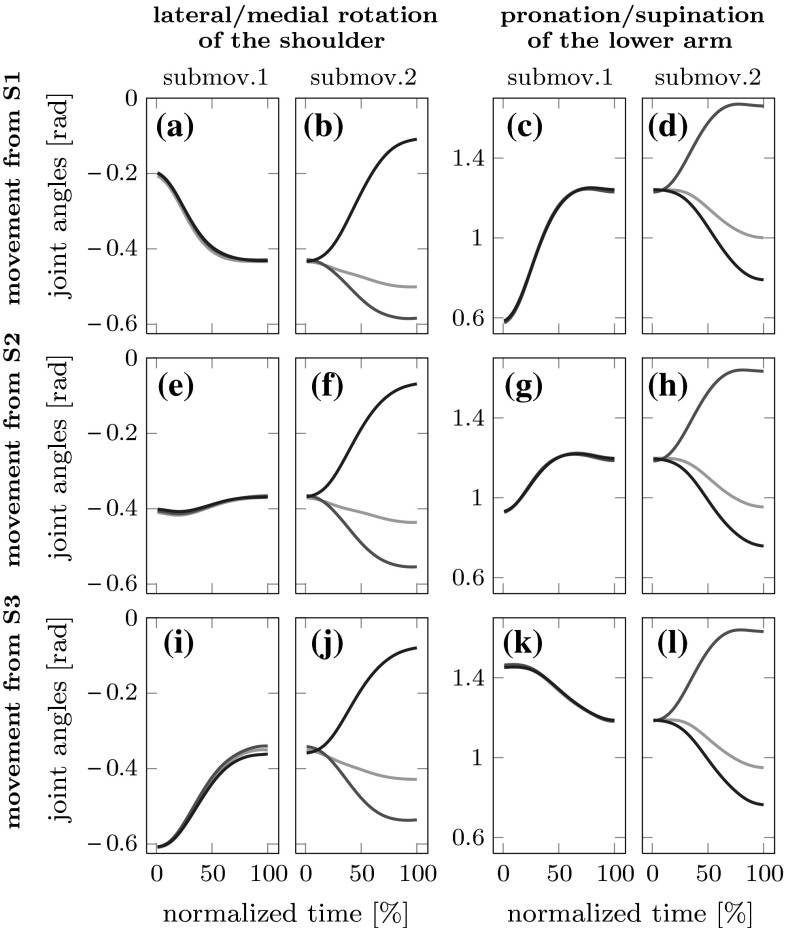
Trajectories of the two joint angles of Fig. 5 plotted for the same movements as in that figure, but now shown separately by starting position (S1, S2 and S3), while trajectories to the different final targets (T2, T4 and T6) are color coded

Figure 6 overlays trajectories of the same two joint angles going to different final target positions, separately for the first three (out of six) starting positions. The second sub-movements lead to different targets, so naturally the final joint configurations differ. However, the trajectories of the first sub-movement are very similar, so that the second sub-movement starts from the same joint configuration and then goes to different targets. This pattern, which is similar for the other three starting positions, suggests the absence of anticipatory coarticulation at the joint level.

Similar patterns consistent with carry-over coarticulation, but not with anticipatory coarticulation, can be observed for other joint angles.

### Motor equivalence analysis to detect coarticulation

How joint angle configurations depend on movement context can be analyzed in a more systematic way using the UCM variant of MEQ. In that approach, we compare the mean joint configuration trajectory observed when a movement is performed after a first segment that started from one position (Si) to the mean joint configuration trajectory when the movement is performed after a first segment that started at a different position (Sj). In the comparison, we compute the difference vector at each point in time and project that vector onto the null-space (UCM), within which the end-effector is invariant, and its orthogonal complement ORT. Because the two subspaces have different dimensionality (three DoFs for ORT, $10-3=7$ DoFs for UCM), we normalize the length of the difference vector in each subspace by the number of DoFs. The two numbers that result are the amount of joint configuration difference that is not motor equivalent (non-MEQ), computed from the ORT subspace, and the amount of joint configuration difference that is motor equivalent (MEQ), computed from the UCM subspace.

**Fig. 7:**
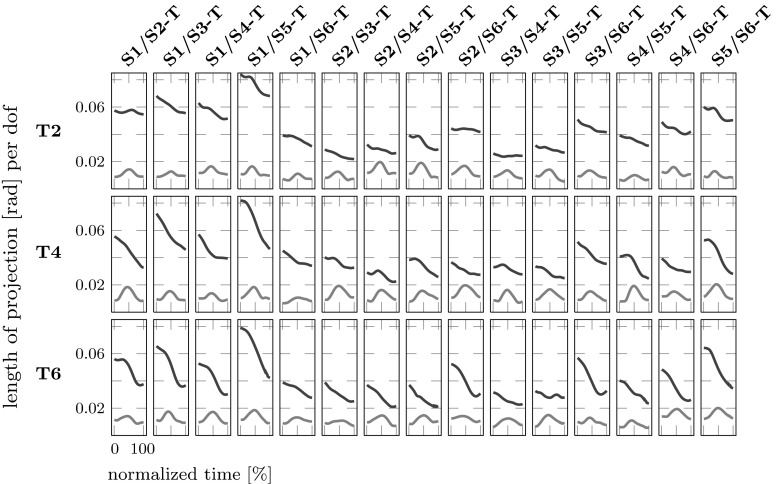
Mean (over all participants) motor equivalent (MEQ, *black*) and non-motor equivalent (Non-MEQ, *red*) components of the joint deviation vector, plotted as functions of normalized time for the second sub-movement. *Rows* refer to the three final targets. *Columns* depict the comparisons across different starting positions labeled on *top*

**Fig. 8:**
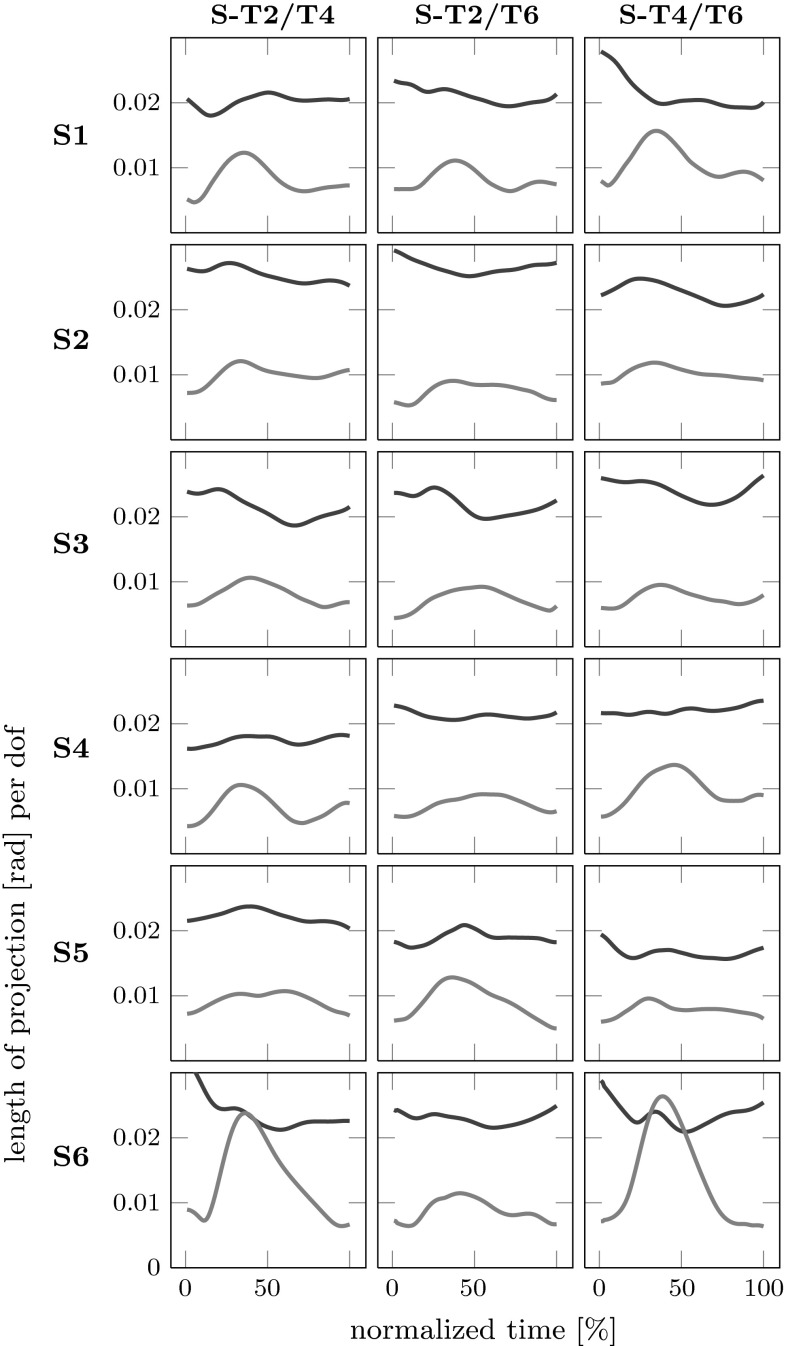
Mean (over all participants) motor equivalent (MEQ, *black*) and non-motor equivalent (Non-MEQ, *red*) components of the joint deviation vector, plotted as functions of time for the first sub-movement. *Rows* refer to the six starting positions. *Columns* depict the three comparisons across different final target positions labeled on *top*

Figure 7 shows the results of the MEQ analysis for carry-over coarticulation. All possible comparisons across different first sub-movements are made for the second sub-movement. The MEQ component always exceeds the non-MEQ by a comfortable margin over the entire second sub-movement. Specifically, the motor equivalent component (MEQ) ranges from 0.02 to 0.062 rad per DoF and for the non-motor equivalent component (non-MEQ) from 0.01 to 0.02 rad/DoF.

However, the results of the MEQ analysis to detect anticipatory coarticulation are different as shown in Fig. 8. All possible comparisons across different second sub-movements are made for the first sub-movement. MEQ differences lie around 0.02 rad per DoF, lower than for carry-over coarticulation. Non-MEQ lies around 0.01 rad per DoF and exceeds MEQ in two cases. Moreover, the MEQ difference varies less over time than in carry-over coarticulation. While MEQ still exceeds non-MEQ, the difference is smaller. High values of MEQ would indicate coarticulation at the level of the joint angles, whereas high values of non-MEQ would indicate coarticulation at the level of the end-effector. High values in both difference measures could, in principle, be observed, indicating coarticulation at both levels. In Figs. 7 and 8, non-MEQ is relatively small, specially at the start and at the end of each sub-movement. A slight increase in non-MEQ in the middle of each sub-movements could be caused by a slight mis-alignment of the trajectories in the two conditions that the measure compares.

In summary, there is no clear evidence for coarticulation at the level of the end-effector. There is clear evidence for carry-over coarticulation at the joint level, but also a suggestion of anticipatory coarticulation at that level. On the other hand, visual inspect of individual joint trajectories (Fig. 6) did not suggest anticipatory coarticulation at the joint level. To resolve this apparent discrepancy, we have a closer look at the distribution of the underlying data. In a nutshell, the MEQ result is contaminated by the UCM variance result. That contamination is strong for anticipatory, but weak for carry-over coarticulation.

**Fig. 9:**
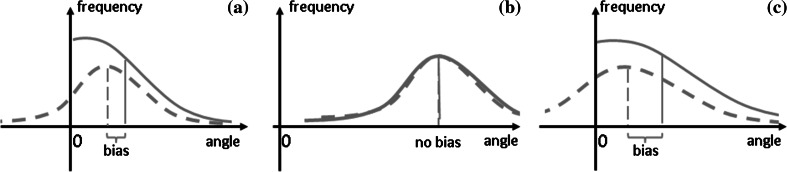
Schematic illustration of a distribution of the joint difference vector $\Delta \theta$ (*dashed line*), here depicted along one dimension of joint space and the resulting distribution of the length of that difference vector (*solid line*). **a** The mean of the length measure is biased for distributions lying close to zero, **b** but not for distributions far from zero, where “close” is relative to the width of the distribution. In **c**, the same mean difference as in panel **a** is shown with larger variance, leading to larger bias for the mean length

The difference between two mean joint configurations, $\Delta \theta$, is computed in the multidimensional joint space, but then assessed by the length of its components in the two subspaces. These lengths are positive numbers. In distributions of positive numbers, the mean and the variance are linked. This is illustrated schematically in Fig. 9, in which we assume, for illustration, that the joint angle difference lies along a single dimension so that the length of the joint angle difference is just the absolute value of the difference. When the mean joint angle difference lies close to zero compared to the variance of the underlying distribution (Fig. 9a), then the mean length of the difference vector is biased toward larger values. Essentially, the contributions of negative values of the joint angle in the distribution pull the mean joint angle difference toward lower values. No such pull toward lower values is possible in the distribution of the positive length measure. When the mean joint difference lies at larger values relative to the variance (Fig. 9b), then no such bias occurs. When the bias occurs, it is larger if the variance of the underlying distribution is larger [panel (c) compared to panel (a) of Fig. 9].

We apply this understanding to the distributions underlying the mean length of the difference of joint configuration in the two subspaces. For anticipatory coarticulation, the length of the mean difference vector in ORT is small (around 0.01 rad/DoF) compared to variance (the SD is of the order of 0.03 rad/DoF), similarly for UCM (around 0.02 rad/DoF for mean length and 0.06 rad/DoF for SD). So the length measures are biased. The bias is larger for UCM, because UCM variance is larger. This may explain the observation of MEQ in the anticipatory coarticulation, which may, therefore, be an artifact of bias.

For carry-over coarticulation, in contrast, the mean lengths of difference vectors are larger (around 0.04 rad in UCM, 0.015 in ORT), closer to the SD of the underlying distribution, leading to smaller bias. The larger variance within UCM will thus have less of an effect, and the observed MEQ effect that supports carry-over coarticulation may be real.

### Statistical analysis of motor equivalence

To assess motor equivalence free of these biases, we statistically analyze the joint angle difference vectors over time directly in the two subspaces UCM and ORT, using these vectors as dependent variables of sets of MANOVAs (multivariate analysis of variance). For carry-over coarticulation, we performed MANOVAs for every participant, the five points in time at 1, 25, 50, 75 and 100 % of the second sub-movement and for each of the three targets. The main factor was the starting position of the first sub-movement with six levels (S1–S6). To analyze anticipatory coarticulation, we performed MANOVAs for every participant, five points in time of the first sub-movement, and each of the six starting positions. The main factor was the target position of the second sub-movement with three levels (T2, T4 and T6). The P values were adjusted using the Bonferroni correction. Alpha was set at $P = 0.05$.

**Fig. 10:**
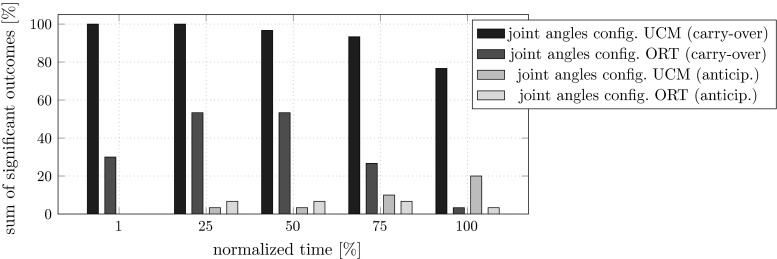
Percentage of MANOVAs that yield significant effects for carry-over coarticulation (*first two bars*) and anticipatory coarticulation (*last two bars*) at five points in time during the movement. The MANOVAs used as dependent variables either the joint configuration difference vector in the UCM space (*first* and *third bar*) or the joint configuration difference vector in the ORT space (*second* and *fourth bar*)

In Fig. 10, we summarize the outcomes of these MANOVAs by reporting the percentage of MANOVAs that led to significant effects for each of the five points in time. The figure shows three outcomes quite clearly. First, the analysis for carry-over coarticulation led to distinctly more significant effects than the analysis for anticipatory coarticulation (first two bars at each point in time compared to last two bars). This meant that the second sub-movement depended on starting position more often than the first sub-movement on the upcoming target. Second, for carry-over coarticulation, there are considerably more significant effects for MEQ than for non-MEQ (first bar at each point in time compared to second bar), meaning that there is more evidence for the preceding movement leading to motor equivalent differences in joint configuration than to non-motor equivalent ones. Third, for anticipatory coarticulation, there are no significant effects at the beginning of the first sub-movement and few at later points in time. Thus, joint configurations only occasionally depended on the upcoming movement.

## Discussion

We observed how participants made two sequential movements, transporting a cylindrical object from a starting to a center position and on to a final target position. The naturalistic movements entailed ten DoF, so that the arm was redundant with respect to the three-dimensional positioning task (the system remains redundant if we assume that orientation of the object along two dimensions was also controlled). We analyzed whether movements depended on the preceding movement (carry-over coarticulation) or on the upcoming movement (anticipatory coarticulation). Overall, we found no hint at either type of coarticulation at the level of the end-effector path. At the joint level, however, we uncovered clear evidence for carry-over coarticulation, but not for anticipatory coarticulation.

### UCM effect of variance

Before we address coarticulation, we briefly discuss the structure of variance across trials. We saw a clear UCM effect for joint configuration variance: There was more variance in those directions in joint space, in which the task variable, hand or object in space, is not affected (UCM), than in directions in which it is affected (ORT). UCM variance exceeds ORT variance at all times (Fig. 2). This is consistent with earlier studies of reaching (Tseng et al. 2002; van der Steen and Bongers 2011; Jacquier-Bret et al. 2009; Mattos et al. 2011). In some of that earlier work, an increase in the variance in ORT in the middle of the movement was observed (Tseng et al. 2003; Scholz et al. 2000). This may arise from variance in the alignment of different trajectories, which translates into variance in joint space when speed is high (most strongly thus for the ORT component that reflects the movement of the end-effector and most pronounced at peak speed in the middle of the movement). In our data, that modulation of ORT variance is barely visible. This may be due to reliable alignment of the trajectories, enabled by the low variance of movement timing. There is a slightly higher UCM variance at the beginning of the first sub-movement that reflects variability in the initial arm posture. The initial posture of the arm was controlled through the spatial location of the object held by the participant. We did not impose an invariant arm configuration. Other studies specified the initial arm configuration and found lower UCM variance at the beginning of the movement (Krüger et al. 2012; van der Steen and Bongers 2011). The homogenous UCM effect across movements and across time also reflects that the arm is moving within a portion of workspace, in which the number of DoF is not constrained by kinematic singularities or joint limits.

### Coarticulation

In the past, coarticulation in arm movements has often been primarily analyzed at the level of the end-effector trajectories. Carry-over coarticulation in a sequence of movements avoiding obstacles was observed, for instance, in the sense that the vertical elevation of the hand over a surface required to pass an obstacle early in the sequence affected the elevation of movements later in the sequence (van der Wel et al. 2007). We have not observed carry-over coarticulation at the end-effector level. Such a negative outcome does not exclude coarticulation at that level, of course. The effect may have been too weak to be detected for the very simple movements that were performed without time pressure.

Anticipatory coarticulation was not necessarily expected in the present experimental paradigm, because participants did not need to attend to the second target before initiating the first movement given that a visual marker remained visible at the location of the second target during the movement. Participants may also not have had sufficient time to attend to the second target. Bullock et al. (1999) found, however, evidence for anticipatory coarticulation at the end-effector level when participant moved the hand from one point to another through a via point. As in the present experiment, both final target and via point were visible through the movement. The movement task required participants to move over the via point, not to stop and touch a surface there, however. Continuity among movement segments may promote anticipatory coarticulation. Similarly, strongly different final arm postures may promote anticipatory coarticulation (Rosenbaum et al. 2006). In the present setting, final postures may not have differed enough to invoke such an effect.

At the joint level, we found indices for carry-over coarticulation based on visual inspection of the joint trajectories. This is broadly consistent with earlier reports according to which final joint configuration of a redundant arm pointing at the same target depends on the starting location (Soechting et al. 1995; Nishikawa et al. 1999). We have found that this dependence on starting location is preserved through a subsequent movement, leading to carry-over coarticulation.

### UCM approach to coarticulation

A more systematic analysis of coarticulation at the joint level makes use of the concept of the UCM. This approach makes it possible to systematically analyze how much of the dependence context, that is, on preceding or subsequent movements, can be attributed to the end-effector and how much resides exclusively at the joint level. The idea is to compute the difference vector between joint configurations observed in the different movement contexts. This difference is decomposed into its component within UCM and within its orthogonal complement, ORT. The size of the difference vector within ORT is a measure of coarticulation at the end-effector level. The size of the difference vector within the UCM is a measure of “pure” joint-level coarticulation.

We found clear evidence for carry-over coarticulation within the UCM. Carry-over coarticulation within ORT was negligible for both carry-over and anticipatory coarticulation. There was a small amount of anticipatory coarticulation within the UCM. By uncovering a subtle interaction between the assessment of coarticulation in the two subspaces and the classical UCM effect in variance, we were able to show that this small effect was spurious. Briefly, when the size of difference vector within each subspace is assessed, a scalar measure must be computed. We used the length of the projected vector, normalized by the dimension of the subspace. Statistically, however, the mean of a distribution of lengths is biased toward larger values because the distribution is restricted to nonnegative numbers. This bias is larger for broader distributions. Because the UCM effect implies that the distribution within the UCM subspace is broader than in the orthogonal complement, the bias is larger for the UCM than for the orthogonal component. We solved this problem by performing multivariate statistics on the length of the difference vectors and thus found that there was no significant anticipatory coarticulation even within the UCM.

### Motor equivalence

We can compare how much coarticulation lies at the end-effector level and how much “purely” at the joint level by comparing the two components of the difference vectors. If more of the difference lies in the UCM than orthogonal to it, then end-effector depends less on context than the joint configuration itself. This was true in our data when movement context was provided by the past (carry-over coarticulation), but not when movement context was provided by the future (anticipatory coarticulation). Making this comparison amounts to asking about MEQ, that is, asking whether the mean join configuration chosen under different experimental conditions is invariant primarily at the level of a task variable or primarily at the level of a particular joint configuration (Scholz et al. 2007, 2011; Schöner et al. 2008; Mattos et al. 2011).

The UCM conception of MEQ thus makes exact the notion we have suggested earlier that there is more coarticulation at the joint level than at the end-effector level. A priori, a statement like this may be problematic. After all, the end-effector is measured in different units (e.g., centimeters) than the joint configuration (e.g., in degrees). Differences in end-effector vs. joint configuration induced by movement context are, therefore, not directly comparable. The UCM conception of MEQ makes such comparison possible by embedding differences in both end-effector and joint configuration in a single space, the space of joint configurations, and measuring the size of the differences induced by context in different directions in that space (per DoF).

The analysis of MEQ used here shares with the UCM analysis of variance, the basic geometrical conception of measurement. In both cases, differences are mapped onto directions in joint space and are thus made comparable. These differences are variances in the classical UCM analysis, while they are differences of the mean configurations between conditions in the analysis of MEQ. Note, however, that the two types of analysis do not necessarily converge. A priori, it would be thinkable for the difference between the joint configurations chosen in different movement contexts to lie perfectly in ORT. This would be the case, for example, if these coarticulation effects were to arise from motor plans that were made in terms of end-effector trajectories. The variance could still lie more strongly in UCM than ORT as it could arise, for instance, from the system that enacts the motor plans, that is, from the system of control. Except for the subtle problem of estimation we uncovered, the mean joint configuration and its variance reflect different processes that do not need to show the same signature.

### Coarticulation and movement planning

So now that we have clearly established carry-over coarticulation that takes place at the joint rather than at the end-effector level, what does this reveal about how movement sequences are planned and controlled? We have also seen that there is little anticipatory coarticulation. Overall, therefore, the end-effector level is more invariant across movement context than the joint level.

One interpretation of this observation is that sequential movements are planned at the end-effector level and that these movement plans do not overlap across the segments of sequences of simple point to point hand movements.

Where does the joint-level carry-over coarticulation then come from? In this interpretation, carry-over coarticulation is a signature of the transformation of motor plans at the end-effector level into motor control signals at the joint level. Given that the arms kinematics is redundant, there is no unique transformation from the end-effector to the joint level. In robotics, the set of possible transformations is described through kinematic pseudo-inverses (Whitney 1969; Liegeois 1977; Klein and Huang 1983). The relevant property of pseudo-inverses is that they do lead to a dependence of the final joint configuration on the initial joint configuration when the end-effector moves from an initial to a final position. When the end-effector moves from different initial positions to a final position, then the initial joint configurations naturally differ. These differences remain in the final position of the end-effector and persist into the subsequent movement. The pseudo-inverses predict, therefore, carry-over coarticulation.

### Pseudo-inverse and self-motion

**Fig. 11:**
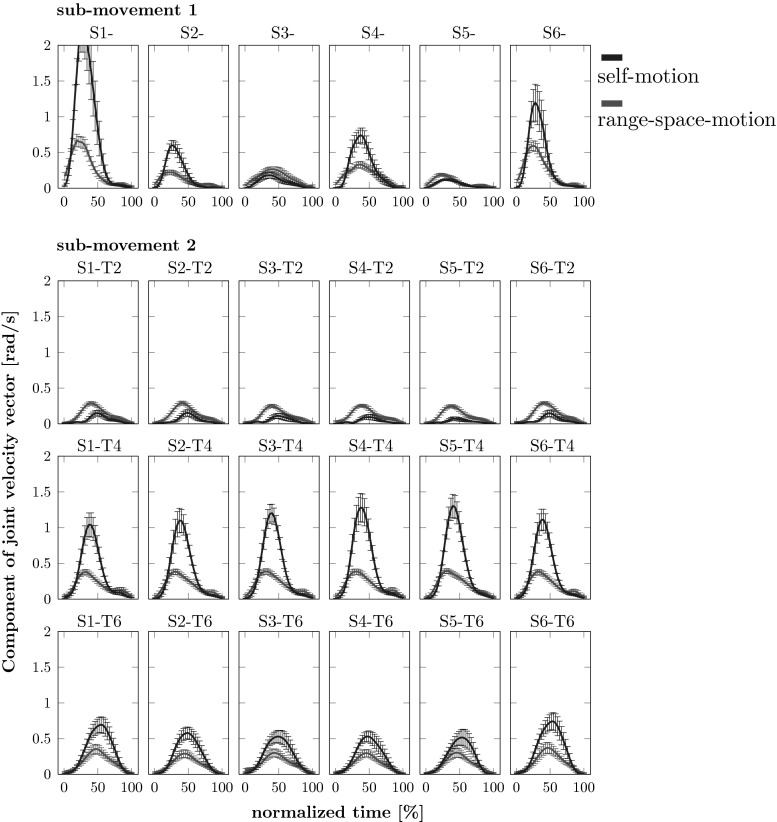
Self-motion and range-space motion are shown as functions of normalized time (means across trials and participants). These were obtained by projecting the joint velocity vectors onto the UCM and the ORT subspaces, respectively, and computing the length per degree of freedom of each component velocity vector. The *first row* shows the first sub-movement coming from the six starting positions (averaged across final target condition). The *bottom three rows* show all second sub-movements from all starting positions to all final target positions

The kinematic pseudo-inverse (also called Moore–Penrose pseudo-inverse) also predicts that the movement of redundant effects should occur with minimal self-motion so that joint velocity vectors are largely orthogonal to the UCM. In fact, such has been directly postulated in models of human movement planning [cf. Rosenbaum et al. (1999); Hollerbach and Flash (1982)]. In Fig. 11, we show that this is not true for our data. When we decompose the joint velocity vector into its components parallel and orthogonal to the UCM, we find a large component within the UCM, that is, a massive amount of self-motion. This seems to refute the explanation of joint-level carry-over coarticulation by kinematic pseudo-inverses.

A more refined analysis can take its departure point from an earlier effort to explain movement in redundant effector systems using pseudo-inverses implemented within neural network models (Brüwer and Cruse 1990; Cruse et al. 1993). The pseudo-inverse accounted for the dependence of joint configurations on the starting position of the end-effector, the first step in our argument above. The authors argued, however, that the pseudo-inverse by itself was not sufficient to account for human movement and pointed to the need to consider the control properties of muscles. A neural dynamic model of movement generation in redundant effectors (Martin et al. 2009) combines a form of pseudo-inverse with dynamic models of the control of muscles (Gribble et al. 1998) that are based on the equilibrium point hypothesis (Feldman 1986). In that model, the starting position will affect the joint configuration used to reach a new target. Counterintuitively, this model also predicts that redundant human movement should exhibit considerable self-motion, a prediction confirmed in human experiment (Martin et al. 2009; Scholz et al. 2011). In the model, self-motion arises even if the *motor plan* is free of self-motion. The self-motion arises because the muscles do not perfectly realize the commanded trajectory.

The model of Martin et al. (2009) is critical to understand the balance of two factors in this study. On the one hand, carry-over coarticulation implies that different histories of the end-effector path are preserved through different joint configurations even when the end-effector movement becomes identical and thus now longer reveals the different past. This is typical of pseudo-inverse kinds of solutions. On the other hand, such differences in joint configurations are bounded by joint limits of the arm or by uncomfortable arm configurations (Rosenbaum et al. 1999). Reducing motor equivalent differences between joint configurations requires self-motion, that is, motion that moves the joint configuration without moving the end-effector. The model of Martin et al. (2009) suggests, therefore, that a movement plan at the end-effector level leads to a plan at the joint level, shows carry-over coarticulation and is, itself, free of self-motion. Motor control by muscles, on the other hand, induces self-motion which limits the amount of carry-over coarticulation by moving joint configurations back toward a more typical reference configuration.

## Conclusion

Our experiments have thus established carry-over coarticulation at the level of the joint angles by applying the UCM concept to MEQ across different movement contexts. Anticipatory coarticulation was not observed nor were any coarticulation effects observed at the end-effector level. This is consistent with the notion that motor plans are elaborated and sequentially organized at the end-effector level. The translation of end-effector motor plans to the joint level has elements of a pseudo-inverse that predicts carry-over coarticulation. The observation of strong self-motion shows that this transformation is more complex, however. The combination of inverse kinematics, neural coupling structure and muscle dynamics of Martin et al. (2009) reconciles the observed signatures of carry-over coarticulation and self-motion.

In this account, the motor plan at the end-effector level is sequential and does not overlap across segments, leading to the absence of coarticulation at the end-effector level. It is possible that tasks that make stronger demands on planning movement sequences as a whole or that put considerable time pressure on executing movement plans will lead to coarticulation also at the end-effector level, including anticipatory coarticulation. To assess coarticulation that comes from movement planning, the present discovery of carry-over coarticulation at the joint level is a relevant constraint, as this form of coarticulation can be accounted for at the level of movement generation and control alone.
